# Tailoring facilitation strategies to enhance learning outcomes in nursing simulations: A practical guide

**DOI:** 10.5339/qmj.2025.89

**Published:** 2025-09-15

**Authors:** Anan Al Badawi, Guillaume Alinier, Abdulqadir J. Nashwan

**Affiliations:** 1 Department of Medical Education, Hamad Medical Corporation, Doha, Qatar; 2 Hamad Medical Corporation, Doha, Qatar; 3 Weill Cornell Medicine-Qatar, Doha, Qatar; 4 School of Health and Social Work, University of Hertfordshire, Hatfield, UK; 5 Faculty of Health and Life Sciences, Northumbria University, Newcastle upon Tyne, UK *Email: aalbadawi1@hamad.qa

**Keywords:** Simulation-based education, Novice to Expert theory, facilitation strategies, nursing, simulation outcomes

## Abstract

**Introduction:**

Simulation-based education (SBE) is an effective teaching method in nursing education that integrates theoretical knowledge with practical application. It provides nursing students with a safe and controlled environment where they can apply their knowledge, develop essential clinical skills, and make critical decisions - without compromising patient safety.

Nursing learners possess varying levels of experience, ranging from novice to expert, as outlined in Benner’s “Novice to Expert” theory. This variation in expertise necessitates the design of simulation experiences that are tailored to accommodate these diverse levels of competence. This practical guide presents evidence-informed strategies to support simulation facilitators in tailoring their facilitation approaches for nurses ranging from novice to expert.

**Methods:**

A narrative conceptual review approach was employed to examine the literature on facilitation in SBE. Key theoretical perspectives, including “Novice to Expert” theory and adaptive approaches, were used to frame the discussion. The review synthesized findings from existing studies and frameworks to develop this practical guide, which supports the use of tailored simulation facilitation strategies in nursing education.

**Results:**

Tailoring facilitation strategies based on learners’ developmental stages enhances learner engagement, performance, and learning outcomes. Novice learners benefit from structured guidance, close supervision, and stepwise instruction. In contrast, proficient and expert learners require facilitation approaches that promote autonomy, critical thinking, and complex decision-making. This guide emphasizes the importance of adjusting simulation facilitation strategy, feedback style, and simulation complexity to meet learners’ needs. Additionally, adherence to established simulation standards supports consistency and effectiveness of facilitation across different learner levels.

**Conclusion:**

Simulation facilitators play an important role in customizing learning experiences based on nursing learners’ level of expertise. Applying Benner’s Novice to Expert theory enables facilitators to provide appropriate support, which enhances the educational outcomes of simulations. Future efforts should focus on faculty development and institutional support to ensure facilitators are prepared to deliver adaptive, learner-centered simulation experiences.

## 1. INTRODUCTION

Simulation plays a crucial role in nursing education by providing immersive experiences that integrate theoretical knowledge with practical applications for nursing students and professionals.^1^ Its significance lies in its ability to provide learners with a safe opportunity to apply their knowledge, develop clinical skills, and make decisions without causing harm to real patients.^2^ Nursing learners differ in their knowledge and expertise, requiring the simulation facilitator to adopt different approaches when facilitating a simulation session and ensure that the complexity of the activity is adapted to match the learners’ needs.^3,4^

Although simulation is widely recognized as a valuable learning tool, its effectiveness can vary depending on participants’ prior knowledge, experience, and skills. Additionally, the same simulation typology may yield different learning outcomes due to variations in how educators facilitate the session, the learning objectives, and how the learners engage with the activity. Therefore, no two simulation-based activities are guaranteed to yield identical results, as outcomes are influenced by both instructional design and learner involvement.^5^ Acknowledging these differences and customizing facilitation approaches allows educators to optimize learning experiences and maximize learning outcomes for nursing learners. The aim of this article is to explore evidence-based strategies for tailoring simulation facilitation from novice to expert nurses, drawing on empirical research and real-world examples to provide actionable insights for simulation facilitators.

## 2. TAILORING SIMULATION FACILITATION APPROACH FOR NURSING LEARNERS

Nurses participating in any simulation-based education (SBE) activity vary in their levels of experience, knowledge, and skills. Moreover, personal attributes, interests, circumstances, experiences, and professional and educational opportunities will emphasize such diversity among individuals. Benner’s “Novice to Expert” theory outlines five levels of nursing proficiency, ranging from novice to expert.^6^ These levels can be effectively applied to learners practicing with SBE. Categorizing nursing learners by their level of expertise enables educators and facilitators to tailor SBE activities to meet the specific needs and learning objectives of each group of learners. The following sections provide an overview of each level, along with recommended facilitation strategies.

### 2.1. Novice

Novice nurses are nursing students or nurses in their first year of clinical practice, during which they often exhibit constrained and rigid behavior within the clinical environment.^6^ Novices demonstrate a limited capacity to predict potential patient deterioration in specific clinical situations. For example, novices are taught about blood pressure, pulse, and other vital signs in an objective manner, without reference to any specific patient. Task features that can be recognized without situational experience are identified. Another example is that alterations in a patient’s mental condition become apparent to novice nurses only after they have encountered other patients with similar symptoms.^6^

Cant and Cooper reported that simulation enhances confidence and acquisition of basic skills in undergraduate nursing students, including physical examination and communication skills.^7^ Clear instructions and defined objectives help novices understand what is expected of them, while immediate feedback is essential for correcting mistakes and reinforcing learning. Moreover, teaching simple and repetitive tasks – such as monitoring vital signs, practicing hand hygiene, and administering medication – can help build their confidence and competence to perform these tasks.^8,9^ As in rapid cycle deliberate practice, the facilitator provides step-by-step instructions, pauses the scenario for immediate feedback, and closely supervises the performance to ensure correct technique and adherence to safety protocols.^10^ Immediate feedback is also given after the task to highlight the areas for improvement and reinforce correct practices.^11,12^ Post-simulation debriefing should be an essential part of the educational process for novice nurses, representing a concept distinct from feedback. While feedback focuses on specific actions and provides immediate corrections, simulation debriefing encompasses a broader and more reflective process. Debriefing provides novice nurses an opportunity to engage in critical thinking and self-reflection by analyzing their performance during the simulation. This reflective practice enables them to understand the rationing behind their actions, consider alternative approaches, and internalize key learning points.

### 2.2. Advanced beginner

Advanced beginner nurses have some experience and can identify recurring meaningful situational components, but they still need help with prioritizing tasks. While they continue to rely on established rules, they begin to recognize important aspects of situations. They may not yet grasp the bigger picture yet but can identify some relevant elements when making decisions.^6^ For example, an advanced beginner nurse might notice that a patient’s blood pressure is low and recall from prior experience that this could signal shock. However, they may not fully understand all the implications or know the appropriate next action to be taken without consulting more experienced colleagues or referring to guidelines.

At this level, learners benefit from moderately complex simulations that incorporate real-life scenarios while still requiring significant nursing care.^6^ They begin to apply learned rules to actual situations. Research indicates that advanced beginners benefit from simulations incorporating diverse patient care situations, enabling them to begin recognizing patterns and applying theoretical knowledge in practical settings.^13^ Encouraging reflection after simulations help the learners to understand the underlying principles guiding their actions. The facilitator can guide the learner through the assessment process, prompting them to consider factors such as vital signs and patient history. Following the simulation, a debriefing session enables the learner to reflect on their decisions and understand the reasoning behind their actions.^11^

### 2.3. Competent

Competence, typically observed in nurses with two to three years of experience, emerges when they begin to view their actions in relation to consciously recognized long-term goals or plans.^6^ They use advanced planning and organizational skills. Competent nurses are able to recognize patterns and assess clinical situations more quickly and accurately than advanced beginners.^6^ Competent learners are prepared for simulations that require critical thinking and decision-making. They benefit from scenarios involving multiple issues that require prioritization of care. Koukourikos et al. highlight that simulation helps competent nurses improve their clinical judgment and serves as a value-added adjunct to their clinical practice, particularly when scenarios are designed to reflect real-world complexities.^14,15^ Facilitators should provide scenarios that require comprehensive planning and multitasking. Competent learners benefit from simulations that challenge their problem-solving abilities and require them to prioritize tasks under pressure. In these situations, the facilitator’s role shifts to that of a mentor – offering less direct supervision and focusing instead on providing feedback regarding the overall performance and decision-making processes.^14^ For example, a simulation for a competent nurse might involve leading a team during a complex emergency scenario, such as managing a multi-trauma patient. The facilitator observes the learner’s ability to coordinate with team members, manage resources, and make quick, informed decisions. The debriefing session may include providing feedback focusing on their leadership skills and effectiveness in implementing clinical protocols.

### 2.4. Proficient

Proficient nurses understand situations as wholes rather than parts because they perceive their meaning in terms of long-term goals. Their learning is characterized by an intuitive grasp of situations based on deep understanding.^6^ This holistic view enables them to consider the broader context of a patient’s condition and often leads them to take on leadership roles to coordinate care effectively. Through experience, they learn to anticipate typical events in a given situation and understand how to adjust their plans accordingly in response to those events.^6^

Proficient nurses benefit from highly realistic scenarios that enable them to practice adapting care plans in response to changes in the patient’s condition within a controlled environment. These scenarios help refine their ability to make decisions and coordinate care effectively, thereby enhancing their holistic approach to patient care.^16^ Facilitators can further challenge proficient nurses by introducing scenarios with unexpected complications and encouraging them to adapt their techniques dynamically.^17^ For example, a proficient nurse might participate in a high-fidelity scenario involving a rapidly deteriorating patient with multiple comorbidities. The facilitator may introduce sudden changes in the patient’s condition, requiring the learner to make timely decisions. After the simulation, a detailed debriefing session helps the learner analyze their decisions and understand how their experience guided their actions.^11^

### 2.5. Expert

Expert nurses at this level can identify the demands and resources required to achieve their goals, relying on their extensive knowledge and experience to guide their actions rather than strictly following rules. They focus on the most important problems, using analytical tools only when encountering unfamiliar events or when situations deviate from expectations.^6^ Expert nurses, drawing on extensive experience, develop an intuitive understanding of clinical situations, allowing them to quickly identify the core of a problem without wasting time on less likely alternatives.^6^ This level of expert performance is difficult to explain because it stems from a deep, often subconscious grasp of the entire situation – much like a chess master or a business leader who justifies a decision by saying it “felt right” or “it all depends.”

Expert nurses benefit from simulations that challenge their diagnostic skills and adaptability. They perform best in scenarios that require innovative problem-solving. Binstadt et al.^18^ found that simulation was beneficial for experienced emergency staff to perform rarely encountered procedures and could be adopted by other institutions with minimal risk to actual patients. To effectively facilitate simulations for expert nurses, strategies should include designing complex and highly realistic scenarios that replicate real-life emergencies, incorporating rare and unfamiliar procedures to practice critical but infrequent skills, and fostering innovative problem-solving through challenging scenarios.^19^ Additionally, reflective practice through debriefing sessions, peer-led simulations that encourage collaborative learning, and the use of advanced analytical tools for detailed performance feedback are essential.^18^

The significance of the Novice to Expert theory lies in its depiction of how nurses progress in learning from relying on abstract principles to mastering concrete experiences. As outlined in this model, novices begin by strictly adhering to rules and guidelines due to their lack of contextual understanding. As they advance through stages, they transition from theoretical understanding to intuitive, experience-based decision-making. This progression emphasizes the role of practice and experience in developing expertise and practical judgments.^20,21^ This theory has reshaped the understanding of what it means to be an expert nurse.^6^

Recognizing the level of nursing learners is crucial for designing effective learning experiences that cater to the individual needs and abilities of each participant. Bahreman and Swoboda highlights the importance of recognizing learner levels in SBE, emphasizing the need for personalized learning approaches to optimize educational outcomes.^22^ Additionally, Cheng et al.^23^ underscore the benefits of tailoring simulation experiences to accommodate different learning styles and preferences, resulting in improved engagement and knowledge retention. Acknowledging and embracing the varying level of nursing learners enables nursing educators to create inclusive learning environments that promote active participation and enhance learning effectiveness in SBE.

## 3. SIMULATION DEBRIEFING FOR NOVICE TO EXPERT NURSING LEARNERS

Effective debriefing is a crucial component of SBE that facilitates reflection and the integration of new knowledge into clinical practice. In SBE, it is essential to incorporate a structured debriefing process, regardless of the learner’s experience level.^24^ Debriefing should be facilitated by a trained individual and aim to promote analysis at the individual, team, and system levels. The primary goal is to guide learners toward a comprehensive understanding of the simulation experience, thereby achieving the intended learning objectives and outcomes.^24^

Debriefing can take place either after the simulation event (“post-event debriefing”) or during the simulation (“within-event debriefing”).^25^ Within-event debriefing, also known as a reflective pause, involves a deliberate interruption at any point during the learning activity to encourage immediate reflection.^26^ Facilitators may plan these pauses in advance or initiate them in response to observed critical behaviors.^26^ Both post-event and within-event debriefing strategies are valuable for learners across all proficiency levels, helping them accurately assimilate and apply their learning in clinical practice. Another approach reported to help learners reflect on their clinical practice and identify personal learning needs – especially post-simulation experience – is the use of a cue card.^27^

Facilitators should plan the debriefing phase in advance and communicate the plan to learners during the pre-briefing session. This preparation ensures that learners are aware of the debriefing process and its role in their learning experience.

## 4. EXPLORING FURTHER APPROACHES TO SIMULATION FACILITATION

LeFlore and Anderson emphasize the detailed approach required in simulation facilitation, a vital component of healthcare education.^28^ The art of delivering simulation sessions involves the following three levels of facilitation by the educator, each tailored to the learners’ experience and needs. This approach can be effectively implemented in nursing education.

### 4.1. Self-directed facilitation

This approach is suitable for expert nurses who can effectively manage the scenarios with minimal facilitator intervention. According to a model that links the level of realism in SBE activities to the type of learner engagement, this self-directed facilitation approach allows learners’ unprompted actions to generate a high level of realism, making it a student-led activity.^29^ In this context, the facilitator’s role is to observe and allow the learners to apply their knowledge and skills independently.^28^ This method promotes autonomy and self-confidence by encouraging the learners to rely on their clinical reasoning and decision-making abilities. Research shows that self-directed learning during scenarios enhances critical thinking and problem-solving skills, making it particularly effective for advanced learners.^30^ Arizo-Luque et al. found that self-directed simulation facilitation helped learners improve in both motivation and critical thinking, showing growth in personal characteristics, cognitive and intellectual abilities, interpersonal skills, self-management, and technical competencies.^31^

### 4.2. Cueing learners

Cueing involves a facilitator or an embedded participant providing subtle hints or prompts to guide learners through the scenario or to bridge the gaps in the simulation fidelity.^19^ This approach is beneficial for nursing learners ranging from novice to competent, who have some experience but may occasionally need support to stay on track or overcome challenges.^28^ Cueing helps to ensure that learners do not become overly frustrated or lost during the simulation, allowing them to remain immersed in the simulation and effectively achieve the learning objectives. Cueing the participants can involve using embedded participants, altering patient parameters, modifying the simulation environment, and the guidance from the facilitator.^32,33^ Jeffries found that cues assist learners in identifying their current step by re-establishing their situational awareness or providing additional information to help them progress in the scenario.^34^

### 4.3. Expert or instructor model

In this approach, the facilitator instructs and intervenes during the scenario. This model is useful for novice nurses who require more direct support and guidance. The educator’s real-time feedback helps learners understand the context and develop their skills more effectively.^28^ This model ensures that foundational concepts are reinforced, and learners can immediately apply feedback to improve their performance. Aebersold and Tschannen highlight that direct instruction during simulation-based activities is crucial for building basic competencies and boosting confidence in beginners.^35^ Kardong-Edgren et al. reported that expert modeling can assist nursing students in recalling and executing a complex series of tasks within a scenario.^36^

## 5. THE ROLE OF ADAPTABILITY IN SIMULATION FACILITATION

Based on the previous discussion about the facilitation approaches, facilitators must be adaptable and capable of modifying their approach according to the participants’ skill levels, learning objectives, and the context in which the simulation occurs. The adaptability is essential for addressing the needs of nursing learners, including their knowledge, skills, attitudes, and behaviors. Moreover, facilitators must be culturally competent, recognizing and respecting individual differences that may impact the learning experience.^37^ This competence ensures that all learners feel valued and supported, thereby enhancing engagement and learning outcomes.

Facilitators play a crucial educational role in SBE by being attentive and adaptive to students’ cognitive and emotional responses to the simulation. The study by Madsgaard et al. highlights the importance of training facilitators to understand the role of emotions in learning and to develop strategies for observing and adjusting their training to address emotional needs.^38^ In addition, a research by Fanning and Gaba emphasizes the importance of adaptability in debriefing sessions, where facilitators must be flexible in addressing learners’ individual learning needs and providing timely feedback.^39^ Adapting debriefing techniques to accommodate different learning styles and preferences allows facilitators to enhance the effectiveness of post-simulation discussions and promote reflective learning.^11,40,41^ Moreover, adaptability is essential for ensuring the relevance and applicability of scenarios to participants’ professional roles and responsibilities. According to Dieckmann et al., educators should modify scenarios to align with participants’ clinical practice settings, patient populations, and specific learning objectives, thereby enhancing the transfer of learning to real-world contexts.^42^

## 6. BEST PRACTICES FOR EFFECTIVE SIMULATION FACILITATION

To effectively design and facilitate simulation sessions, best practice recommendations include conducting a thorough needs assessment, adopting a participant-centered approach, and providing robust debriefing sessions.^21,43^ A needs assessment helps identify the learners’ specific skills and knowledge gaps, enabling for the design of targeted simulations. A participant-centered approach involves engaging practitioners in the design process, ensuring that the simulations are relevant to their work environments and professional responsibilities.^44^

The “Healthcare Simulation Standards of Best Practice™ Facilitation” outlines guidelines to ensure the effectiveness of SBE in healthcare.^45^ These standards emphasize the key role of facilitators in guiding participants through simulations, highlighting the need for them to possess the appropriate education, skills, and abilities to lead effectively when needed. Facilitators are essential in helping participants develop critical thinking, problem-solving abilities, clinical reasoning, and judgment skills – crucial for applying theoretical knowledge to patient care in various healthcare settings.^45^ The following sections outline the “Healthcare Simulation Standards of Best Practice™ Facilitation”, presenting essential guidelines to ensure the effectiveness of SBE in healthcare.

### 6.1. Facilitator education and skills

Facilitators must demonstrate a robust understanding of simulation pedagogy, integrating both theoretical and practical knowledge to enhance learning experiences. They need to be adept in selecting and implementing facilitation methods that align with the learners’ needs and the intended learning outcomes. The ability to select the appropriate method is crucial for optimizing the educational impact of simulations.^45^ In addition, continuous professional growth is essential for effective facilitation. Facilitators must engage in ongoing education and skill assessments to stay current with best practices in SBE.^46^ This ongoing development ensures facilitators adapt to new techniques and technologies, maintaining the relevance and efficacy of their teaching methods. Regular training and workshops can help facilitators stay abreast of the latest developments in simulation technology and pedagogy, ensuring they provide the best possible learning experiences for participants.^45,47^ To ensure the quality and effectiveness of SBE, facilitators’ competencies should be regularly evaluated and aligned with institutional goals and accreditation standards to support quality assurance and maintain educational excellence. This responsibility partly falls on the simulation center or program’s leadership, who oversee the assessment of facilitators’ skills and their ongoing professional development. Using structured evaluation tools like the Facilitator Competency Rubric – which assesses key domains such as preparation, pre-briefing, facilitation, debriefing, and evaluation – offers a comprehensive framework for this process.^48^ In addition, facilitators are expected to pursue external recognition of their simulation education competence through certification or accreditation, as now offered by several relevant societies.^21^

### 6.2. Creating a safe learning environment

A key aspect of effective facilitation is creating a psychologically safe environment that encourages learning and reflection during and after the scenario. This involves establishing a learning space where nursing learners feel comfortable to make mistakes and learning from them without the fear of judgment. According to Kang and Min, psychological safety is essential for participants to fully engage in simulations and achieve meaningful learning outcomes.^49^ Facilitators must be skilled in fostering this environment, which includes setting clear expectations, providing supportive feedback, and encouraging open discussion. Facilitators should start the pre-briefing by clearly outlining the goals and expectations of the simulation session, emphasizing that the purpose is to learn and improve rather than to perform perfectly.^21^ This sets a tone that values growth and understanding over perfect execution.

During debriefing, facilitators should focus on constructive feedback that highlights both strengths and areas for improvement in a manner that encourages – rather than discourages – participants. It is important to remind learners that the primary objective of the scenario is learning. Encouraging open discussion is another crucial component of the debriefing. Facilitators should invite participants to share their thought processes, decisions, and emotions experienced during the simulation.^11^ They should listen carefully to participants’ concerns and address any negative reactions resulting from the simulation experience. This approach not only helps nursing learners articulate their reasoning but also fosters a culture of transparency and trust.^24^

### 6.3. Supporting skill development and critical thinking

Effective facilitators play a crucial role in the development of essential skills and cognitive abilities in nursing learners. They guide participants through the simulation process, providing support and feedback to help them achieve the desired competencies. For example, facilitators can use debriefing techniques to help participants reflect on their performance, recognize areas for improvement, and consolidate their learning. This reflective practice is essential for developing higher-order thinking skills and enhancing the learners’ clinical reasoning and judgment skills.^41,45^

Adhering to these standards is essential to ensure the effectiveness of SBE. When facilitators are well-prepared and skilled in their roles, the simulations are more likely to be engaging and educationally valuable. Conversely, failing to adhere to these standards can impair participants’ engagement and reduce the effectiveness of the SBE experience. For example, insufficient facilitator competence or inadequate preparation can result in poorly executed simulations that fail to meet educational objectives, affect the debriefing process, and potentially result in negative learning experiences.^21^

## 7. CHALLENGES AND SOLUTIONS IN SIMULATION FACILITATION

### 7.1. Common challenges in tailoring simulation facilitation

Tailoring the simulation facilitation to accommodate nursing learners with varying abilities presents several challenges. The primary challenge is the varied baseline knowledge and skills among participants. While novice nurses may struggle with basic concepts, advanced learners might find fundamental scenarios unchallenging and unengaging. This disparity can hinder effective learning and skill acquisition among a diverse group of learners.^51^

The second challenge is the resource intensity required for tailored simulations. Highly realistic simulations require substantial financial, technological, and human resources to accurately replicate clinical encounters, which can be a limiting factor – especially for underfunded institutions.^52^ Additionally, the design and development of custom simulations require expertise and time, which further strains resources.^51,53^

The third challenge is related to the facilitator’s expertise. Skilled facilitators must be adept at adjusting their teaching strategies to accommodate different learning needs and provide appropriate feedback or facilitate an effective debriefing depending on the type of activity.^11^ Lastly, evaluating the effectiveness of tailored simulations can be complex. Developing metrics that accurately reflect the learning outcomes for different participant levels is difficult, and without robust evaluation, it is challenging to ensure that the simulations are meeting their educational needs.^54^

### 7.2. Strategies to overcome challenges

Various strategies can be implemented to overcome the challenges of simulation facilitation. One effective approach is to employ a modular design for scenarios. This involves creating core scenarios with optional advanced components that can be included or excluded depending on the participants’ skill levels, ensuring that all are appropriately challenged and engaged. Additionally, tailoring scenarios based on evidence-based needs assessments is essential for ensuring a meaningful learning experience.^44^ The needs assessment guides facilitators to develop learning objectives that match the learners’ knowledge and expertise, thereby enhancing the simulation’s relevance and impact.^21^ By combining modular design with targeted needs assessments, the facilitators can create flexible and effective simulation experiences that cater to the diverse capabilities of advanced participants.

To address the resource intensity challenge of highly realistic tailored simulations, institutions can adopt a hybrid approach that combines lower-cost, lower-fidelity simulations with targeted high-fidelity elements for critical aspects. Additionally, they can leverage shared resources through partnerships with other institutions, and employ existing simulation templates to reduce development time and expertise requirements – thereby optimizing resource use without compromising educational quality.^55^ Investing in facilitator training is essential. Facilitators should engage in ongoing professional development to enhance their ability to deliver tailored simulations and provide constructive feedback.^46,47^ Simulation centers can support this by providing workshops and certification programs to ensure facilitators are prepared to manage diverse learning needs.^45^

For evaluation, employing a multi-faceted approach can provide a comprehensive assessment of learning outcomes. This includes using direct observation, feedback surveys, and performance metrics to measure the effectiveness of the simulations across various participant levels.^54^

Finally, fostering a collaborative environment where educators, technologists, and clinicians work together can significantly improve the development and implementation of tailored simulations. Such multidisciplinary collaboration ensures that simulations are comprehensive, technically robust, and clinically relevant, thus enhancing their educational impact.^45^

## 8. CONCLUSION

Simulation can be an effective educational approach for developing essential clinical competencies in the nursing profession. Nurses vary in their expertise, requiring tailored simulation experience and facilitation strategies. This practical guide contributes to the state of the art in simulation-based nursing education by offering a structured, evidence-based guide for educators to tailor facilitation strategies according to learners’ proficiency levels, based on Benner’s “Novice to Expert” theory. It explores the integration of facilitation strategies (self-directed, cueing, and instructor-led) with learner levels, supported by empirical evidence and real-world examples. It also emphasizes psychological safety, cultural competence, and facilitator adaptability – critical underrepresented elements in the simulation literature. In the future, this guide can inform the design of modular simulation curricula and faculty development programs. It can also guide future research on the impact of tailored facilitation on SBE outcomes.

## COMPETING INTERESTS

The authors have no conflicts of interest to declare.
